# LipidFinder on LIPID MAPS: peak filtering, MS searching and statistical analysis for lipidomics

**DOI:** 10.1093/bioinformatics/bty679

**Published:** 2018-08-07

**Authors:** Eoin Fahy, Jorge Alvarez-Jarreta, Christopher J Brasher, An Nguyen, Jade I Hawksworth, Patricia Rodrigues, Sven Meckelmann, Stuart M Allen, Valerie B O'Donnell

**Affiliations:** 1 Department of Bioengineering, University of California San Diego, San Diego, CA, USA; 2 Institute of Infection and Immunity, School of Medicine, Cardiff University, Cardiff, UK; 3 Babraham Institute, Babraham Research Campus, Cambridge, UK; 4 School of Computer Science and Informatics, Cardiff University, Cardiff, UK

## Abstract

**Summary:**

We present LipidFinder online, hosted on the LIPID MAPS website, as a liquid chromatography/mass spectrometry (LC/MS) workflow comprising peak filtering, MS searching and statistical analysis components, highly customized for interrogating lipidomic data. The online interface of LipidFinder includes several innovations such as comprehensive parameter tuning, a MS search engine employing in-house customized, curated and computationally generated databases and multiple reporting/display options. A set of integrated statistical analysis tools which enable users to identify those features which are significantly-altered under the selected experimental conditions, thereby greatly reducing the complexity of the peaklist prior to MS searching is included. LipidFinder is presented as a highly flexible, extensible user-friendly online workflow which leverages the lipidomics knowledge base and resources of the LIPID MAPS website, long recognized as a leading global lipidomics portal.

**Availability and implementation:**

LipidFinder on LIPID MAPS is available at: http://www.lipidmaps.org/data/LF.

## 1 Introduction

Recently, liquid chromatography (LC) separation and mass spectrometry (MS) detection of solvent-extracted biological samples has evolved such that thousands of lipid components can be detected and separated in the mass/time domains. Approaches such as high resolution MS, combined with long chromatographic runs enable discrimination of many thousands of lipid ions, something that is not possible using gold standard quantitative MS/MS methods. This offers unparalleled opportunities in the precision medicine space to discover new bioactive species either as biomarkers of disease, or new therapeutic targets. Reliably identifying putative candidate lipids from large lipidomic datasets that change with phenotype is a complex task. Until the recent release of LipidFinder (O’Connor *et al.*, 2017), there was a lack of openly available bioinformatics tools that aimed to reliably remove non-lipid artifacts from the dataset, leaving lipid-like features (Cappadona *et al.*, 2012; O’Donnell *et al.*, 2014). Existing tools such as MZmine (Katajamaa *et al.*, 2006) and XCMS (Smith *et al.*, 2006), originally designed to curate metabolomic datasets, do not adequately tackle the specific difficulties involved in processing lipidomic datasets. LipidFinder addresses this, centering on a unique algorithm that uses a set of heuristics to identify lipid-like peaks while discarding noise and contaminants. The problem of contaminants in MS datasets is significant, including spurious signals that can arise from diverse sources, including common contaminants, adducts, in-source fragments, etc. LipidFinder is designed to work primarily as an add-on to pre-processing tools, focusing on the clean-up of MS data files which have already been pre-processed for peak alignment and integration. Removal of these artifacts results in significantly cleaner datasets that perform better in downstream statistical analysis pipelines. Extensive LC chromatography is essential with the LipidFinder approach to separate isobaric lipids which are a major complicating issue in lipidomics MS. This method is not suitable for ‘shotgun’ applications.

Here we present LipidFinder as a web application on LIPID MAPS, a global online resource and database of lipid structures (Fahy *et al.*, 2011; Sud *et al.*, 2012), along with new innovations including the ability to conduct statistical analysis prior to interrogating a series of 5 databases. Although the LipidFinder peak filtering algorithm can significantly reduce the complexity of a lipidomics dataset, the number of features generated from high-resolution MS instruments can still be quite daunting. Leveraging statistical analysis to focus on those features that significantly change across experimental conditions (e.g. treated versus untreated samples) results in a much smaller peaklist which may be subsequently searched against MS databases and then subjected to more rigorous analysis by retention time or ion mobility as orthogonal parameters.

## 2 Results

**Fig. 1.**
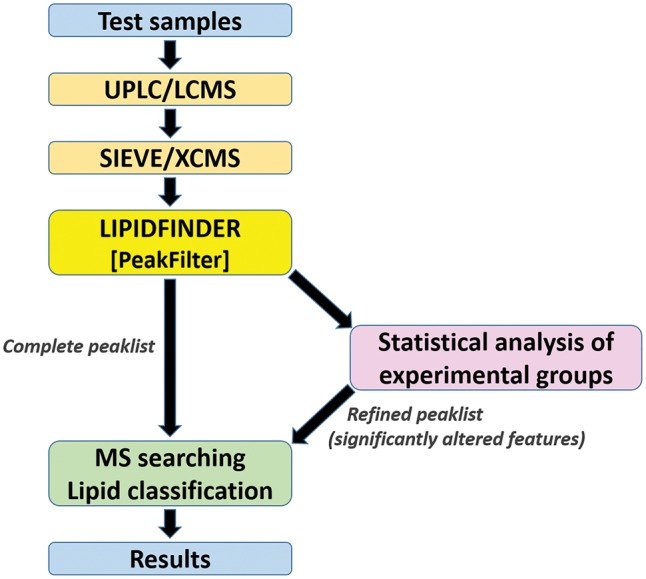
LipidFinder online workflow. Once the file is processed by LipidFinder’s PeakFilter module, users have the option of running a set of statistical analyses to refine the peaklist prior to MS searching. Move this so just under the figure

LipidFinder’s web application follows a similar workflow to its standalone counterpart, as it is outlined in Figure 1. The overall strategy is focused on filtering out a wide variety of artifacts, contaminants and potential false-positives which tend to plague LC/MS analyses of lipids. The end-user is given great flexibility with regard to adjusting peak filtering parameters, MS database searching, lipid classification and statistical analysis of experimental groups. The input data file is generated by XCMS raw data-processing programs that can align chromatograms, correcting RT shifts, and can pick out signals, assigning *m/z* and time ‘features’, combining data from several samples into a single consolidated file. While LipidFinder appears to share similar functionalities with XCMS (mass clustering, feature finding, retention time correction), these use different algorithms in XCMS, and therefore they perform differently to LipidFinder’s versions. We provide on LIPID MAPS examples of how running these functionalities again in LipidFinder significantly improves the quality of XCMS datasets. Additionally, LipidFinder has extra functionalities specifically designed to improve artifact removal that are not in XCMS, including: contaminant, adduct and stack removal, mass reassignment and outlier correction.

This file is uploaded as a comma-separated values (CSV) formatted text file via the web browser interface. The user can then set the parameters for the various data-processing steps based on the experimental conditions deployed through an intuitive interface. If necessary, the user can also upload customized files containing contaminants, adducts and lipid stacks in order to replace the default values used by LipidFinder.

The first stage executes LipidFinder’s PeakFilter module, which utilizes a multi-step algorithm to perform blank and background removal, adduct removal, contaminant removal, lipid stack removal, retention time correction and outlier correction. A detailed description of each process is described elsewhere (O’Connor *et al.*, 2017). In the current version we have only included the PeakFilter module for the data-processing part. Although a key stage of LipidFinder’s performance, the Optimizer module has not been included due to high computational cost. Users can obtain and implement this off-line from GitHub (https://github.com/ODonnell-Lipidomics/LipidFinder).

One innovation of the online version of LipidFinder in contrast to its standalone counterpart is that, on completion of the peak filtering stage, a MS search interface is presented to the user with a choice of five in-house lipidomics and metabolomics databases:

1. **COMP_DB**: a computationally generated database composed of over 30 000 bulk (isobaric) species covering 30 lipid classes which is customized for precursor ion searching.
2. **ALL_LMSD:** the entire LIPID MAPS structure database (LMSD) of over 40 000 discrete lipid structures.
3. **CURATED_LMSD:** a subset of approximately 20 000 curated structures in LMSD which have been reported in the literature and which does not include computationally generated structures.
4. **MET:** a set of over 21 000 metabolite structures in the Metabolomics Workbench metabolite database (Sud *et al.*, 2016) from which all lipids with a LIPID MAPS identifier have been removed.
5. **REFMET:** a set of over 11 000 exact metabolite structures and isobaric species containing both lipids and non-lipids which are used as a MS reference set for the Metabolomics Workbench project.

The user can specify a mass tolerance value, one or more ion adducts to search and optionally may restrict the search to one or more lipid categories or classes, depending on the context. Results may be downloaded as a CSV report file or viewed in the browser as a hyperlinked results table containing relevant structural information, or a merged report with the corresponding retention time and sample intensity data.

Finally, the web application offers a novel alternative where the processed peaklist may be subject to statistical analysis to first identify significantly changed features across the experimental conditions deployed. The user first defines two or more experimental groups for each sample in the dataset (e.g. treated, untreated; wild-type, mutant; timepoints A, B, C; etc.) and is then presented with an interface to four different programs: volcano plot analysis, orthogonal partial least-squares discriminant analysis, random-forest analysis and ANOVA. These methods have the ability to identify features that significantly change across experimental conditions, thereby condensing the input data down to a much smaller peaklist which may be subsequently searched against the MS databases discussed in the previous step.

## Funding

This project was supported by a Wellcome Trust grant for the LIPID MAPS project (203014/Z/16/Z) and the European Research Council (LipidArrays). V.B.O. holds a Royal Society Wolfson Research Merit Award.

*Conflict of Interest*: none declared.
